# Infectious Complications in Chronic Lymphocytic Leukemia

**DOI:** 10.4084/MJHID.2012.070

**Published:** 2012-11-05

**Authors:** Annamaria Nosari

**Affiliations:** Divisione di Ematologia, Niguarda Ca’ Granda Hospital, Piazza Ospedale Maggiore 3 - 20162 Milano, Italy. Tel: 39-02-64442668, Fax: 39-02-64442033.

## Abstract

Infectious complications have been known to be a major cause of morbidity and mortality in Chronic Lymphocytic Leukemia (CLL) patients who are prone to infections because of both the humoral immunodepression inherent to the hematologic disease and to the immunosuppression related to the therapy. The majority of infections in CLL patients treated with alkilating agents is of bacterial origin. The immunodeficiency and natural infectious history of alkylator-resistant, corticosteroid-treated patients appears to have changed with the administration of purine analogs, which has been complicated by very severe and unusual infections and also more viral infections due to sustained reduction of CD4-positive T lymphocytes. The subsequent introduction of monoclonal antibodies in therapies, in particular alemtuzumab, further increased the immunodepression, increasing also infections which appeared more often in patients with recurrent neutropenia due to chemotherapy cycles.

Epidemiological data regarding fungal infections in lymphoproliferative disorders are scarce. Italian SEIFEM group in a retrospective multicentre study regarding CLL patients reported an incidence of mycoses 0.5%; however, chronic lymphoproliferative disorders emerged as second haematological underlying disease after acute leukemia in a French study on aspergillosis; in particular CLL with aspergillosis accounted for a third of these chronic lymphoproliferative diseases presenting mould infection.

## Introduction

Infectious complications have been known for many years to be a major cause of morbidity and mortality in Chronic Lymphocytic Leukemia (CLL) patients. Indeed, they account for the leading cause of death in most series.^1^ Infectious mortality ranges between 30–50%.^2^

Patients with CLL are predisposed to infections because of both the humoral immunodepression related to stage and duration of CLL, and to a further immunosuppression related to therapy with steroids, cytotoxic drugs and monoclonal antibodies.

Encapsulated bacteria (*Streptococcus pneumoniae, Haemophilus influenzae*) are the predominant pathogens in patients with this disease, but in neutropenic post-chemotherapy phase also *Staphilococcus aureus* and various Gram-negative enteric patogens such as *Pseudomonas aeruginosa, Escherichia coli* and *Klebsiella pneumoniae* can be responsible for bacteraemia and septicemia, especially in patients with hypogammaglobulinemia.^3^

*Herpes simplex* and *zooster* are the usual viruses found particularly in advanced disease.^4^

Regarding the main localizations, pneumonia is more severe and frequent, particularly in advanced disease; in neutropenic patients bacteriaemia and septicemia are common. Other common sites of infection include all the respiratory tract, the urinary tract, skin and soft tissue. The immunodeficiency and natural infectious history of alkylator-resistant, corticosteroid-treated patients appears to have changed with the administration of purine analogs,^5^ which has been complicated by severe and unusual infections due to sustained reduction of CD4-positive T lymphocytes. The following introduction of monoclonal antibody therapies, in particular alemtuzumab, probably further increased the immunodepression, increasing also fungal infections, which appeared more often in patients with recurrent neutropenia due to chemotherapy cycles and sporadically after rituximab. Mycoses, in particular cryptococcal meningitis (2.4%), were reported in CLL patients.^6^ Some reports showed sporadical cases of *Candida* and *Aspergillus* infections, which may be related to therapies resulting in more prolonged periods of neutropenia, in patients with advanced disease and/or CD4+ < 200/mL.

## Epidemiology

Epidemiological data regarding fungal infections in lymphoproliferative disorders are scarce; the incidence of mycoses reported by Italian SEIFEM group in a retrospective multicentre study regarding new CLL patients admitted to hospital was 0.5%;^7^ as expected in patients with new diagnosis at different stages of disease, the number of fungal infections was very small (only five cases), however presenting a high attributable mortality (80%). A recent prospective study regarding febrile events in hematologic patients,^8^ showed that only 2 cases of mycoses (moulds) among 172 new CLL patients were diagnosed, confirming the rarity of fungal infections at first diagnosis of this haematological disease. However, chronic lymphoproliferative disorders emerged as second haematological underlying disease (21.6%) after acute leukemia (34.6%) in a French epidemiological study on aspergillosis published in 2011;^9^ in particular CLL with aspergillosis accounted for 30.6% of these chronic lymphoproliferative diseases presenting mould infection.

Similar results were showed also from other studies.^10^–^11^ In particular the PATH Alliance registry^11^ very recently presented epidemiological data regarding 960 patients with aspergillosis; among them CLL patients accounted for 7.1% of the entire haematologic population (464 patients, 48.2%) with aspergillosis.

In our previous experience regarding infectious episodes in 379 CLL patients observed from 1984 to 2002^12^ bacterial infections accounted for 67%, viral for 25% and fungal for 7%, of whom only 3% were severe. The main risk factor for infections seemed to have received multiple lines of chemotherapy.^12^–^13^ No further epidemiological data regarding mycoses are available in CLL patients submitted to multiple lines of chemotherapy, even if in salvage studies fungal infections are seen more frequently, but sporadically. Among them also *Pneumocistis jirovecii* infections were reported as sporadic cases.^14^

Mycobacterial infections (*Mycobacterium tubercolosis* or *atypical mycobacteria*) have been reported rarely, especially in patients with a previous history of infection, because in CLL patients the use of steroids scheduled in chemotherapy cycles or prescribed for autoimmune episodes ( immune haemolytic anemia and/or thrombocytopenia) is an important risk factor for this infection. Mycobacterial infection prevalence in CLL patients was 88 cases/ 10.000 patients.^15^

## Pathogenesis of Infection

The pathogenesis of infection in CLL is multifactorial (Table 1), with both alterations due to primary disease process and immunosuppression by subsequent treatments; however, particularly hypogammaglobulinemia is predictive of an increased frequency of infection.

### CLL-Related Risk Factors

#### Hypogammaglobulinemia

In CLL patients the prevalence of hypogammaglobulinemia varies from 10 to 100% and it is related to duration and stage of the disease.^16^ Reduced immunoglobulin synthesis is probably due to the cell-cell contact with malignant B cells and release of cytokines with inhibitory activity. Usually hypogammaglobulinemia is not reversible even if complete remission is obtained; a recent study has suggested that high doses of rituximab can restore, in part, the production of immunoglobulins.^17^ Low levels of immunoglobulins have been associated with the frequency of infections in these patients, suggesting that the control of CLL reduces also the severity of infections. CLL patients with hypogammaglobulinemia usually show bacterial infections, often recurrent like patients with primary hypogammaglobulinemia. Despite numerous reports correlating hypogammaglobulinemia and infection in CLL, the relationship between the level of a specific immunoglobulin class and the risk of infection is not well-established. Patients with hypogammaglobulinemia may not have infections and, on the contrary, patients with CLL and with normal Ig levels can be subject to recurrent infections; in fact, as well as low Ig levels, it is important that B lymphocytes are able to form a specific response^18^ which is always defective in these patients. Recurrent bacterial infections from *Streptococcus* and *Haemophilus* are particularly associated with low levels IgG.

#### Cell-mediated immunity

Cell-mediated immunity is altered in CLL patients, but from the literature it is difficult to understand if this is a primary defect, strictly related to hematologic disease, or always chemotherapy-induced. Although the absolute T-lymphocyte count is usually normal, reduced T-cell colony forming capacity, an increase in the percentage of T suppressor lymphocytes, a reduction of T-helpers with the reversal of CD4/CD8 ratio and a dominant Th2 response are seen. The CLL cells are capable of inducing changes in CD4+ and CD8+ cells through direct cell-cell contact via inhibitory cytokines such as TNF, IL-6, IL-10 and TGF-beta (19). Also defects in natural killer (NK) cell activity have been reported.^20^ In addition, a poor delayed hypersensitivity response of CLL patients to a variety of skin test antigens such as *Candida albicans*, mumps, and diphtheria toxoid has been found.

#### Neutropenia, neutrophil function and complement

The absolute neutrophil count and neutrophil function are usually normal in untreated CLL patients; neutropenia becomes pathogenetically more important in advanced disease and with more intensive chemotherapy utilization, predisposing these patients to bacterial and fungal infections. Neutrophils and monocytes are defective in their phagocytic function as well as in migration and chemotaxis.^21^

Enzyme deficiency (beta-glucuronidase, lysozyme, myeloperoxidase) was found in neutrophils of some, but not all, patients.^22^ These defects may resolve with hematologic remission.

Heath et al.^23^ showed reduced serum complement levels in CLL patients. Complement plays a crucial role in the control of some bacterial infections; opsonization with complement is necessary for subsequent interactions with neutrophils. It was seen that a low level of C3b fraction of complement was found in the sera of patients with a history of bacterial infections rather than in the sera of patients who had no prior infections.

#### Length of disease

The risk of infection usually increases with the length of disease, in relation to the natural history of the same and to previous therapies undertaken; both these factors can reduce immunoglobulin level. The risk of a severe infection is 26% at 5 years; the risk increases to 57% in patients with profound hypogammaglobulinemia, and to 68% in patients with profound hypogammaglobulinemia and Binet C stage of disease.^2^

#### Advanced stage of disease

There is a significant correlation between the stage of disease and infections ; infectious episodes are not only more frequent, but also more severe in patients in stage C (82%) rather than stage A (33%).^16^

Patients with stage C are a selected group predisposed to infections, in which the severe defect of immune response plays a critical role for infectious morbidity and mortality.

### Treatment-Related Factors

#### Alkilating agents - related infections

Infections are the major cause of death in CLL patients. In a large 24-years observational study of M.D. Anderson Cancer Center 30% of patients died because of infection;^24^ however, this retrospective study was performed to verify different drug combinations and not to investigate the origin of infections and the cause of death; reported data often don’t permit correct evaluation of infectious episodes and presence of correlation with hypogammaglobulinemia, neutropenia, previous treatments and stage of CLL; moreover, they reported data of a selected population with advanced or refractory disease, which is typical of third level centres, to whom more complicated cases are admitted. Despite these limitations, it is evident that the incidence of infections in CLL patients is higher than in the general population, and it develops parallel to the progression of the haematologic disease.

In a review of 518 patients treated with chlorambucil alone, fludarabine alone or with both drugs, chlorambucil-treated patients had fewer major bacterial infections and viral infections than fludarabine treated patients.^25^

In more recent randomized trials,^26^–^28^ grade 3 and 4 neutropenia and grade 3 and 4 infections were reported in 12–28% and in 0–9% of subjects, respectively. In particular when 193 elderly patients (more than 65 years, median age of 70 years) were first-line treated with chlorambucil compared with fludarabine, it showed significant less myelotoxicity but not less incidence rate of severe infections or infections in general (32% vs 26%, P=0.4), even if lethal infections were 3 (3 pneumonias) in the fludarabine arm in comparison with one lethal infection (septic shock ) in chlorambucil arm.^28^

Opportunistic infections are very rare in patients treated with chlorambucil, and only if multiple lines of chemotherapy had been previously administered. A sporadical report of disseminated atypical mycobacteriosis was reported in a CLL patient chronically treated with both prednisone and chlorambucil.^29^

Bendamustine is an alkilating agent recently introduced in the treatment of CLL and able to induce a high number of remissions. In a trial of first-line therapy regarding 319 CLL patients, bendamustine was shown to be more effective than chlorambucil.^27^ Hematologic events and in particular neutropenia occurred more frequently in the bendamustine arm ( 27% vs 14% in the chlorambucil arm); severe infections grade 3 or 4 were present in 8% of the patient treated with bendamustine compared to 3% of patients treated with chlorambucil (only one infection grade 4). Infections occurring during bendamustine treatment may be related to transient neutropenia, and are prevalently bacterial like in utilization of chlorambucil and differently from purine analogs, in which an important cause of infection is due to the prolonged T-cell depletion. Regarding heavily pretreated refractory/relapsed CLL patients, bendamustine was evaluated in a phase I/II study in 16 patients,^30^ showing good efficacy but the initial dose had to be de-escalated due also to severe infectious toxicity. Recently bendamustine combined with rituximab in 78 relapsed/refractory CLL patients^31^ resulted to be safe, showing severe neutropenia in 23% of patients, and grade 3 severe infections in 12.8% of patients, without grade 4 infections. No opportunistic infections were seen.

## Purine Analogs - Related Infections

The discovery of the purine analogs heralded a new era of CLL therapy. Fludarabine, cladribine and pentostatin have all been shown to be active in CLL, inducing up to 20% of complete response in a pivotal trial;^27^ however, their utilization was accompanied by a different spectrum of infections because of selective T-cell abnormalities which these agents determine, especially in heavily pretreated patients. Among these infections, mycoses occupy an important place not so much for their number, but for their high mortality.^6^

Fludarabine is very active against indolent lymphoid neoplasms; its efficacy is due to its ability to reduce the number of lymphoid cells rapidly, originating also some cases of tumor lysis syndrome.

Profound and prolonged suppression of the CD4 cell count occurs, with median CD4 cell counts decreasing <200/mL in 2–3 months of therapy. Although the CD4 cell count improves in the first 3 months from the end of treatment, quantitative abnormalities may persist for 1–2 years. This immunosuppression and neutropenia secondary to therapy determined an increased number of infections, particularly by opportunistics, also in the absence of neutropenia or steroid therapy. Despite the reduced CD4 cell count, the haematologic and clinical response to purine analogues increases macrophage cell activity and often also hypogammaglobulinemia; for this reason infections are more frequent at the beginning of the disease and decrease with improvement of CLL.

In a review of the literature^32^ related to fludarabine-associated opportunistic infections which evaluated 2,269 patients with low-grade malignancies who received fludarabine therapy, the most notable infectious complications were respiratory tract infections and unexplained fever; 3.2% of these patients developed opportunistic infections during or after fludarabine treatment; 97% of these infections occurred in patients who were previously treated with alkylator agents or corticosteroids. Opportunistic infections were due to *Pneumocystis jiroveciii* (33%), other mycoses (30%), *Listeria monocytogenes* (14%), also after many months from completion of therapy, *mycobacteria* (9%), *CMV* (7%), H*erpes* (6%). The majority of these infections doesn’t appear to be related to neutropenia or low levels of immunoglobulins, even if neutropenia in our experience seems to increase the risk of mould infections.

In more recent years three randomized trials in previously untreated CLL patients (33–35) showed that combined chemotherapy with fludarabine and cyclophosphamide (FC) was superior to fludarabine alone. However, hematologic toxicity was higher than with the monotherapy, but no difference was observed in severe infections by fludarabine alone compared with FC.^33^,^35^ In all these trials no fungal infections were observed. Increase of infections was seen in refractory CLL patients treated with combination therapy with fludarabine and cyclophosphamide used as salvage therapy,^36^ which conferred a leukemia survival advantage over patients treated with fludarabine alone; in this population neutropenia occurred in over 50% of cases and significant infection in 48% of patients. Six atypical infections were noted, including one case of *Pneumocystis jirovecii* pneumonia, one cryptococcal bronchitis, one cryptococcal meningitis, one *Vibrio* sepsis, one *Strongyloides*, and one *CMV* infection.

Cladribine, another purine analog, was used alone or combined with other agents in CLL patients with efficacy similar to fludarabine. In a recent trial comparing cladribine plus cyclophosphamide with fludarabine plus cyclophosphamide^37^ also adverse effects were similar; the most common grade 3 and 4 complications were infections which interested the two arms of the study in a similar way (27% in the cladribine arm vs 28% in the fludarabine arm respectively), showing no differences in infectious problems and mortality.

### Monoclonal Antibodies-Related Infections

#### Rituximab

Rituximab is an anti-CD20 monoclonal antibody directed against CD20 antigen present on CLL cells. After rituximab all patients show a rapid decrease in B-lymphocytes, while T-cells remain stable; B-lymphocytes return to normal levels usually within 12 months. Addition of the CD20 monoclonal antibody rituximab to chemotherapy for CLL has improved outcomes, particularly in early lines of therapy. The activity of single-agent rituximab in CLL is modest at standard doses with ORR from 15% to 25%. The major impact of targeting the CD20 has been shown in combination with conventional chemotherapy. When rituximab was added to chemotherapy regimens in chronic lymphoproliferative disorders, infectious complications were comparable to chemotherapy-alone regimens. In particular when this monoclonal antibody was added to standard fludarabine regimen at usual dosage of 375 mg/mq/day, infection did not show an increase.^38^ Similar results were obtained when rituximab was added to fludarabine and cyclophosphamide and compared with the same combination without rituximab;^39^ a better efficacy of the rituximab combination therapy was linked to safe results; however, neutropenia and leukocytopenia occurred more often in chemoimmunotherapy group ( neutropenia 34% vs 21% and leukopenia 24% vs 12% respectively), even if severe or opportunistic infections were not increased with fludarabine, cyclophosphamide and rituximab. This immunochemotherapy resulted more toxic when performed in refractory /relapsed CLL patients. In fact in the M.D. Anderson experience^40^ myelosuppression was the most common reason for discontinuing treatment before completing the courses and occurred in 26% of patients with 6% stopping for infection; neutropenia with grade 3 and 4 was noted in 21% and 41% of cases respectively. Major infections, defined as sepsis, pneumonia or infection requiring hospitalization, occurred in 16%, FUO in 10% and minor infections in 18% of treated patients.

In the current use of rituximab unusual infections, in particular viral infections, have been rarely seen, such as some cases of progressive multifocal encephalitis,^41^ a rare demyelinating disorder of the central nervous system, caused by reactivation of the ubiquitous JC virus.

#### Alemtuzumab

The humanized monoclonal antibody alemtuzumab binds to CD52 antigen, which is present in B and T lymphocytes, monocytes, macrophages and eosinophils, but not in progenitor cells.

Single-agent alemtuzumab showed good efficacy both in first-line therapy and in relapsed/refractory patients and also in combination therapy. However this monoclonal antibody is frequently accompanied by serious complications, in particular cytopenia and subsequent infections. The profound and long-lasting lymphocytolytic activity determined by alemtuzumab is responsible for severe and prolonged immunodepression which produces a major predisposition also to opportunistic infections. In fact, the risk and the type of infection could be different according to indications and doses; its association with chemotherapy, the concomitant administration of immunosuppressive drugs, the characteristics of CLL itself can greatly change the severity of immunodepression, favouring those particular opportunistic infections associated with a low CD4 cell level.^42^ A rapid decrease in normal and malignant lymphocytes is readily apparent within a few weeks of alemtuzumab administration, with T-cells depression after 4 weeks of treatment. Some studies have shown that infections occur at the time of T-cells and neutrophils nadir, while no late-recurring opportunistic infections or major infections were reported during unmaintained, long-term follow-up, probably because the patients are treatment-naïve before alemtuzumab administration.

In a first-line study comparing alemtuzumab with chlorambucil,^43^ although grade 3 and 4 neutropenia was more frequent in alemtuzumab arm, grade 3 and 4 non-CMV infections were similar in the two groups (4.8% with alemtuzumab vs 2.7% with chlorambucil for febrile neutropenia; 3% vs 1.4% respectively for sepsis).

Infectious complications were more frequent in patients who were heavily pretreated or fludarabine-refractory and who did not respond to the same alemtuzumab therapy.^44^ In an earlier phase II study of Osterborg et al.^45^ in 29 previously treated patients with advanced CLL the main side-effects of alemtuzumab treatment were infections, which were related to long-lasting lymphocytopenia. Localized H*erpes simplex virus* reactivation was noted in 11 patients and oral candidiasis in five. Other infections in this trial, which did not incorporate antimicrobial or antiviral prophylaxis, included *Pneumocystis jirovecii* pneumonia (n=2), bacterial pneumonia (n=4) and septicemia (n=3). All patients recovered. A higher percentage of infectious episodes were confirmed also in the pivotal trial using alemtuzumab, which included 93 patients treated at 21 centres in USA and Europe^46^ who had experienced treatment failure from previous therapy with fludarabine and who had also received alkylating agents. Infections occurred in 51 (55%) patients during the study, being mild to moderate in 26 and grade 3 to 4 in 25 cases. Septicemia occurred in 14 patients (15%), and two cases led to death. *Herpes simplex* was present in 6 cases. *CMV* reactivation in 7 patients caused concern. Eleven patients, all of whom with advanced disease, developed opportunistic infections during treatment and further 7 in the follow-up period (1 *Pneumocystis jirovecii* pneumonia, 3 aspergillosis, 1 mucormycosis, 1 cryptococcal pneumonia, 1 *Listeria* meningitis, 4 *Herpes zoster* and 7 *CMV* reactivation). Six of 9 deaths were due to infections. Responders seemed to experience fewer infections than non-responders. Similar results were obtained in another study^47^ regarding patients with relapsed or fludarabine-refractory CLL in which 42% of infections, predominantly in non-responder group, was reported.

Various chemoimmunotherapy combination regimens including alemtuzumab are currently becoming more common therapeutic choices for patients with CLL. Translation of single-agent alemtuzumab into combination regimens has led to variable efficacy but also alteration of the anticipated infectious toxicity profile. Among the combination regimens fludarabine in combination with alemtuzumab (FluCam regimen) was first given to refractory CLL patients,^48^ showing that most infections occurred within the first 3 months, when CD4+ cells were at the lowest level. In particular the patients who had more severe infectious events were those who experienced progressive disease after FluCam treatment. A recent phase 3 study comparing FluCam vs alemtuzumab alone in previously treated patients^49^ showed that all-cause infections occurred in 41% of patients in combination group versus 35% of alemtuzumab group with a similar incidence of grade >3 of infectious toxicity (19 vs 17 cases) in both groups. This initial combination provided the foundation for a number of other chemoimmunotherapy combinations, fludarabine cyclophosphamide and alemtuzumab (FCCam) and fludarabine cyclophosphamide alemtuzumab and rituximab (CFAR) with different schedules.^50^ Safety data^51^ showed an increased incidence of opportunistic infections (but not other types of infections) in the FCCam combination regimen. In a more recent study 52 evaluating cyclophosphamide, fludarabine, alemtuzumab and rituximab as salvage therapy for heavily pretreated CLL patients, 37/80 patients (47%) experienced a serious infection during therapy, while there were 28% of late serious infections in the evaluable patients, showing good responses but no benefit in survival outcome.

Alemtuzumab is also administered as consolidation treatment to clear minimal residual disease. The study of Montillo and colleagues^53^ showed an improvement of quality of response (CR rate from 25% to 79% respectively) administering low dose alemtuzumab (10 mg SC 3 times per week) after fludarabine-based induction. Episodes of CMV reactivation were successfully treated with oral ganciclovir. No bacterial or fungal infections were documented.

However, a recent CALGB study reported severe infections in patients treated with alemtuzumab as consolidation therapy after induction with fludarabine and rituximab.^54^ In particular among 57 treated patients there were 5 deaths in patients who obtained CR resulting from infection (viral and *Listeria* meningitis, *Legionella*, *CMV* and *Pneumocystis jirovecii* pneumonia), which occurred up to 7 months after the last therapy. The study was amended to exclude CR patients to receive alemtuzumab.

In all studies the problem of CMV reactivation related to alemtuzumab administration is recurrent, due to loss of CD8 cytotoxic T cells after lymphocyte-depleting treatment regimens. While CMV reactivation is relatively high in CLL patients treated with alemtuzumab (30–40%), symptomatic CMV infection is rarer and appears in 15% in first–line therapy^43^ and more often in relapsed/refractory patients. Monitoring CMV viremia or antigenemia during alemtuzumab therapy is indispensable for early treatment of symptomatic infection.

In summary, the characteristics and the stage of hematologic disease, in particular refractory disease, play an important role for determining an increase of alemtuzumab-related infections, in particular opportunistic infections. Instead a good response of malignancy to induction therapy such as a less aggressive administration schedules of consolidation seem to determine a lower risk of infection.

### Ofatumumab

Ofatumumab is a fully humanized antibody targeting a distinct epitope on the CD20 molecules of CLL cells. It shows more potent complement-dependent cytotoxicity than rituximab, also in cells with low CD20 expression levels, including CLL cells. In an international multicentre study^55^ ofatumumab was evaluated in 138 patients refractory to fludarabine and alemtuzumab obtaining a good objective response (50%) also in patients who received prior rituximab therapy or combination therapy with FCR. The most common adverse events occurring during treatment were infections (67%) and infusion-related reactions (64%), but the infections related to ofatumumab therapy were 20%. Neutropenia accounted for 19% and fever for 8%; although median neutrophil count decreased during the first weeks of treatment, they remained greater than 1.5 x 10^9^/L and stable during the course of treatment. Among 37 grade 3 and 4 infections, pneumonia (14 cases) and other respiratory tract diseases (6 cases) were the most common. Thirteen patients died due to infections, including sepsis (6), pneumonia (5), *Fusarium* mycosis (1), and progressive multifocal encephalopathy (1), which occurred in a patient with low CD4 count at baseline. Finally the incidence of major infections compared favourably with that of similar patients treated with other salvage regimens (56), which showed up to 60% of major infections and 13% of early deaths and emphasizing the need for prospective evaluation of optimal infection prophylaxis in heavily pretreated patients.

Ofatumumab was also used as front-line therapy in untreated CLL patients in a randomized, 2-doses phase 2 trial^57^ comparing ofatumumab 500 mg with 1000 mg in combination with FC (O-FC), demonstrating to be an active frontline therapy for these patients. Myelosuppression was the most frequent grade 3 or 4 adverse event with this combination therapy, with grade 3 or 4 neutropenia reported in 48% of patients. The mechanism for increased neutropenia with the addition of CD20 monoclonal antibody is unknown, but was independent of the ofatumumab dose level. Infections were present in 38% of administrations; in particular grade 3 and 4 infections accounted for 3% in 500 mg ofatumumab arm and 13% in 1000 mg arm respectively. These infection rates compared favourably with the 26% incidence reported with the FCR regimen.

## Prophylactic Strategies

The prevention of infections is very important in CLL patients undergoing innovative therapies.

Prevention of *Pneumocystis jiroveci* pneumonia in pretreated patients undergoing fludarabine and/or alemtuzumab is of current use, because the incidence of pneumocystosis can be as high as 7% in this patient population.^58^ As in HIV patients with a CD4 cell count less than 200 cells/mL sulfamethoxazole-trimethoprim (one double-strength tablet three times a week) became the treatment of choice in prophylaxis of these patients at least until 2 months after discontinuation of fludarabine treatment and for at least 6 months after completion of alemtuzumab treatment. Sulfamethoxazole-trimethoprim is effective also against listeriosis and other bacterical infections, is inexpensive and relatively well tolerated and it is suitable for long-term use.

The administration of prophylactic intravenous immunoglobulin is not recommended to be used as routine. HD Ig should be performed only in selected cases of severe, recurrent bacterial infectious episodes due to encapsulated bacteria^59^ and requiring hospital admission; in a randomized study, low doses Ig (250 mg/Kg every four weeks) were equivalent to higher doses (500 mg/Kg) in defending from severe bacterial infections.^60^

Considering that only small series or sporadic cases of mycobacterial infections have been described,^15^ prophylaxis against mycobacterial organisms should be considered only if previous history of this infection is present.

Acyclovir is active against *Herpes* viruses and is usually performed as prophylaxis in patients treated with fludarabine and /or monoclonal antibodies (400 mg twice daily), particularly in patients with previous herpetic infections (800 mg twice daily).

Reactivation of *CMV* should be suspected and carefully monitored with antigenemia and/or PCR during and after alemtuzumab treatment, particularly in patients who are *CMV* seropositive at baseline; only symptomatic infection should be treated. Patients presenting with FUO should be tested promptly, also if negative at baseline. In symptomatic cases ganciclovir (5 mg/kg BID) should be performed as first-line treatment for 14–21 days and alemtuzumab suspended until negative *CMV* status is obtained.

General antifungal prophylaxis is not recommended. However, antifungal prophylaxis with fluconazole should be considered, only if mucositis and fungal colonization are present, or during prolonged neutropenia episodes. *Aspergillus* spp have been reported only sporadically in patients treated with both fludarabine and alemtuzumab,^61^ in particular if neutropenic.

The suboptimal response to vaccination in CLL patients may be related to impaired antibody production. Anti-pneumococcal vaccination should be recommended, also in presence of a reduced response. The response of CLL patients to influenza vaccine has also been studied;^62^ after influenza vaccination mean antibody titers were low, but sufficient especially in the early stages of this hematologic disease. Reimmunization with influenza vaccine at 1 month could be necessary because of decrease in antibody titers.

### Conclusions

Knowledge of the range of infections (Table 2) and increased risk in patients treated with multiple lines of chemotherapy (particularly with purine analogs and/or alemtuzumab) or with advanced disease and above all neutropenic enables a prompt institution of surveillance and prophylaxis, when indicated, and a correct therapeutic approach to infection.

## Figures and Tables

**Table 1:** Pathogenesis of infection in CLL patients

**CLL-RELATED RISK FACTORS**
***Hypogammaglobulinemia***
***Cell-mediated immunity*** (T-cells, delayed hypersensitivity)
***Neutropenia, neutrophil function and complement.***
***Length of disease***
***Advanced stage of disease***
**THERAPY-RELATED RISK FACTORS**
***Steroid-induced immune defects***
***Purine analogs, alemtuzumab – induced T-cell defects***
***Neutropenia***

**Table 2:** Spectrum of related-therapy infections

AGENTS	INFECTIONS
**Alkilators*(chlorambucil, bendamustine)***	**Common bacterial pathogens***(Streptococci, Staphylococci, enteric GRAM-negative bacteria)*
***Purine analogs***	**Herpesvirus, opportunistic infections***(Candida, moulds, Pneumocystis jiroveci)*
***Monoclonal antibodies***	
**Rituximab**	***Sporadic viral infections***
**Alemtuzumab**	**Herpesvirus, in particular CMV, opportunistic infections***(Candida, moulds, Pneumocysis jiroveci)*
**Ofatumumab**	**No change in spectrum of infection**
